# Plasma-Coated Polycaprolactone Nanofibers with Covalently Bonded Platelet-Rich Plasma Enhance Adhesion and Growth of Human Fibroblasts

**DOI:** 10.3390/nano9040637

**Published:** 2019-04-19

**Authors:** Svetlana Miroshnichenko, Valeriia Timofeeva, Elizaveta Permyakova, Sergey Ershov, Philip Kiryukhantsev-Korneev, Eva Dvořaková, Dmitry V. Shtansky, Lenka Zajíčková, Anastasiya Solovieva, Anton Manakhov

**Affiliations:** 1 Scientific Institute of Clinical and Experimental Lymphology—Branch of the ICG SB RAS, 2 Timakova str., 630060 Novosibirsk, Russia; svmiro@yandex.ru (S.M.); leravalera0204@mail.ru (V.T.); permyakova.elizaveta@gmail.com (E.P.); 2 Institute of Biochemistry – subdivision of the FRC FTM, 2 Timakova str., 630117 Novosibirsk, Russia; 3 Laboratory of Inorganic Nanomaterials, National University of Science and Technology “MISiS”, Leninsky pr. 4, 119049 Moscow, Russia; kiruhancev-korneev@yandex.ru (P.K.-K.); shtansky@shs.misis.ru (D.V.S.); 4 Physics and Materials Science Research Unit, Laboratory for the Physics of Advanced Materials, University of Luxembourg, 162a, avenue de la Faïencerie, L-1511 Luxembourg, Luxembourg; sergey.ershov@uni.lu; 5 CEITEC—Central European Institute of Technology—Masaryk University, Kamenice 5, 625 00 Brno, Czech Republic; evke.dvorakova@gmail.com (E.D.); lenkaz@physics.muni.cz (L.Z.)

**Keywords:** polycaprolactone, nanofibers, COOH plasma, cell adhesion and spreading, cell viability, freeze–thawed platelet-rich plasma immobilization

## Abstract

Biodegradable nanofibers are extensively employed in different areas of biology and medicine, particularly in tissue engineering. The electrospun polycaprolactone (PCL) nanofibers are attracting growing interest due to their good mechanical properties and a low-cost structure similar to the extracellular matrix. However, the unmodified PCL nanofibers exhibit an inert surface, hindering cell adhesion and negatively affecting their further fate. The employment of PCL nanofibrous scaffolds for wound healing requires a certain modification of the PCL surface. In this work, the morphology of PCL nanofibers is optimized by the careful tuning of electrospinning parameters. It is shown that the modification of the PCL nanofibers with the COOH plasma polymers and the subsequent binding of NH_2_ groups of protein molecules is a rather simple and technologically accessible procedure allowing the adhesion, early spreading, and growth of human fibroblasts to be boosted. The behavior of fibroblasts on the modified PCL surface was found to be very different when compared to the previously studied cultivation of mesenchymal stem cells on the PCL nanofibrous meshes. It is demonstrated by X-ray photoelectron spectroscopy (XPS) that the freeze–thawed platelet-rich plasma (PRP) immobilization can be performed via covalent and non-covalent bonding and that it does not affect biological activity. The covalently bound components of PRP considerably reduce the fibroblast apoptosis and increase the cell proliferation in comparison to the unmodified PCL nanofibers or the PCL nanofibers with non-covalent bonding of PRP. The reported research findings reveal the potential of PCL matrices for application in tissue engineering, while the plasma modification with COOH groups and their subsequent covalent binding with proteins expand this potential even further. The use of such matrices with covalently immobilized PRP for wound healing leads to prolonged biological activity of the immobilized molecules and protects these biomolecules from the aggressive media of the wound.

## 1. Introduction

During the last decade, a lot of effort was focused on the research and development of biodegradable polymer materials for medical applications. The advantage of synthetic biodegradable polymer matrices is the lack of immunogenicity, the numerous possibilities for their modification, the controllability of their mechanical properties, and the rate of decomposition. This allows the creation of materials with the necessary properties for a wide range of applications in regenerative medicine. Both cellular and cell-free materials are being developed. Cell matrices demonstrate greater efficacy for the treatment of deep and chronic wounds [1,2,3,4]. Matrices for cell colonization must have the structure of an extracellular matrix (ECM), which provides structural support and intercellular contact, serves as a reservoir for signaling molecules, and thereby regulates cell migration, proliferation, and angiogenesis [1,2].

The fibrillary, porous structure of the polycaprolactone nanofibers obtained by the electrospinning method mimics the ECM structure [5,6]. However, their surface is hydrophobic and unsuitable for cell viability. In order to increase the bioavailability of polycaprolactone (PCL) nanofibers, plasma treatment [7,8], the deposition of plasma coatings [9], biomineralization [10,11], and the grafting of silk fibroin [12] are used. It is worth noting that the electrospinning of PCL nanofibers is very versatile and the addition of nanoparticles into the electrospinning solution can induce antibacterial properties [13].

The effectiveness of cell matrices depends on the functional activity of the cells used, which in turn depends on the initial processes of adhesion and spreading on the matrix. Cell adhesion to a functionally well-founded substrate is characterized by a well-defined time, as well as mechanical and energy-dependent processes, determining further cell proliferation, cell migration, cell differentiation, and gene expression [14,15,16,17]. It should be noted that the spreading of cells on the surface of substrates is a complex process and it is controlled by the matrix–integrin interactions that form adhesion sites [15,18,19,20,21]. The co-spinning of PCL with collagen [22,23], gelatin [24], chitosan [25], or other bioactive substances [26,27] enriches matrices by natural adhesion sites, thereby improving the cell adhesion properties of nanofibers and consequently contributing to the normal cell functioning [17,18]. Platelet-rich plasma is often used in regenerative medicine because it is a naturally balanced ensemble of growth factors, components of the extracellular matrix including fibronectin (FN) necessary for dynamic connection with substrate [14,28,29,30,31,32].

Fibroblasts are most often used in tissue engineering [33]. These cells are characterized by high plasticity and, depending on the conditions, their functionality changes. The secretion of ECM components (fibronectin, collagens, elastin, laminin, hyaluronic acid, proteoglycans) is one of the main properties of fibroblasts, and their nature depends on the quality of the substrate.

In this work, the morphology of PCL nanofibers was optimized by tuning the needle-less electrospinning process parameters, whereas the chemistry of the PCL nanofibers was activated by the plasma deposition of a COOH layer. This study determined the effect of the modification of PCL nanofibers by the COOH plasma polymerization and freeze–thawed platelet-rich plasma immobilization on the adhesion and spreading of fibroblasts, as well as their proliferative potential and viability. It was shown that the effective immobilization of COOH groups, followed by the covalent bonding of freeze–thawed platelet-rich plasma, led to a physiological course of adhesion and spreading processes, resulting in good viability, proliferation, and contact inhibition of proliferation when the monolayer is reached by the cells.

## 2. Materials and Methods

### 2.1. Preparation of Freeze–Thawed Platelet-Rich Plasma

The methodology of preparation of freeze–thawed platelet-rich plasma (PRP) was described in [34]. In brief, platelet-rich plasma was taken from healthy donors and, subsequently, it was activated using three freeze–thaw cycles. Then, PRP was centrifuged at 12,000× *g* for 10 min at 4 °C. The supernatant containing growth factors was recovered and kept frozen at −70 °C until later steps. The study was approved by the Ethics Committee of the RICEL—branch of ICG SB RAS (Identifier: N115 from 24 December 2015).

### 2.2. Electrospining of Nanofibers

The electrospun nanofibers were prepared by the electrospinning of solution with different concentrations of PCL. A description of the processing of samples can be found elsewhere [5]. The granulated polycaprolactone (80,000 molecular weight (mw)) was dissolved in a mixture of acetic acid (99%) and formic acid (98%). All compounds were purchased from Sigma Aldrich (Darmstadt, Germany. The weight ratio of acetic acid (AA) to formic acid (FA) was 2:1. The concentration of PCL was varied from 7 to 12 wt.%. The PCL solutions in AA and FA were stirred for 24 h at 25 °C. The PCL solution was electrospun with a 20-cm-long wired electrode using a Nanospider™ NSLAB 500 machine (ELMARCO, Liberec, Czech Republic). The applied voltage was in the range from 30 to 60 kV. The distance between the electrodes was set at 100 mm. The as-prepared PCL nanofibers are referred to as PCL-ref throughout the text.

### 2.3. Deposition and Characterization of Plasma Polymer Layers

The COOH plasma polymer layers were deposited using the vacuum system UVN-2M equipped with a rotary and oil diffusion pumps. The residual pressure of reactor was below 10^−3^ Pa. The plasma was ignited using a radio frequency (RF) power supply Cito 1310-ACNA-N37A-FF (Comet, San Jose, CA, USA) connected to the RFPG-128 disc generator (Beams & Plasmas, Zelenograd, Russia) installed in the vacuum chamber. The duty cycle and the RF power were set to 5% and 500 W, respectively.

CO_2_ (99.995%), Ar (99.998%), and C_2_H_4_ (99.95%) were fed into the vacuum chamber. The flows of the gases were controlled using a Multi Gas Controller 647C (MKS, Andover, MA, USA). The flow rates of Ar, CO_2_, and C_2_H_4_ were set to 50, 16.2, and 6.2 sccm, respectively. The pressure in the chamber was measured by a VMB-14 unit (Tokamak Company, Oxfordshire, UK) and D395-90-000 BOC (Edwards, Heinenoord, The Netherlands) controllers. The distance between the RF electrode and the substrate was set to 8 cm. The deposition time was 15 min and it led to the growth of ~100-nm-thick plasma coatings. The plasma-coated PCL nanofibers are referred to as PCL-COOH throughout the text.

The surface of the samples was imaged by scanning electron microscopy (SEM) Tescan LYRA3 (Tescan, Brno, Czech Republic) in secondary emission mode (5–10 kV acceleration voltage, working distance 8–10 mm). The samples were coated with a 10-nm-thick gold film deposited by RF magnetron sputtering prior to the imaging in order to avoid charging of surface. The Gwyddion 2.29 software (Brno, Czech Republic) was used for the determination of the diameter of polymer nanofibers from SEM micrographs. The diameters were determined by averaging of values obtained for 30 single independent filaments present in the micrographs taken at the magnification of 10,000×.

The chemical composition of sample surfaces was determined by X-ray photoelectron spectroscopy (XPS) using an Axis Supra spectrometer (Kratos Analytical, Manchester, UK) equipped with a monochromatic Al Kα X-ray source. The maximum lateral resolution of the analyzed area was 0.7 mm. The spectra were fitted using the CasaXPS software after subtracting the Shirley-type background. The binding energies (BE) for all carbon and nitrogen environments were taken from literature [5,9,35]

The water contact angle (WCA) of the nanofibers surface was determined by a contact angle analyzer (KRÜSS EasyDrop DSA20, Hamburg, Germany). Plasma-treated and untreated samples were cut into 1-cm^2^ mats. Then, 5-µL droplets of deionized water were deposited in random locations on the surface and the water contact angle was measured. All the measurements were completed in triplicate. The infrared spectrum in the range from 370 to 4000 cm^−1^ was measured using a Fourier-transform infrared (FTIR) spectrophotometer (Bruker Vertex 80v, Billerica, MA, USA) using attenuated total reflectance mode (ATR-FTIR).

### 2.4. Coating of Scaffolds with PRP

Prior to immobilization of PRP, all samples were sterilized under ultraviolet (UV) light for 45 min. At first, the adsorption of PRP by PCL-ref was investigated. The PCL-ref was immersed in the PRP solution at 25 °C for 15 min and then it was thoroughly washed with phosphate-buffered saline (PBS). The sample was denoted as PCL-PRP. Then, the ionic bonding of PRP to the plasma-coated PCL nanofibers (PCL-COOH) was investigated. The PCL-COOH was immersed in the solution of PRP for 15 min and then it was washed with PBS. This sample was denoted as PCL-COOH-PRP1. In order to achieve covalent bonding of PRP to the PCL-COOH surface, the latter was immersed in a 1-ethyl-3-(3-dimethylaminopropyl) carbodiimide (EDC) (Sigma Aldrich, St. Louis, MO, USA, 98%) solution in water (2 mg/mL) for 15 min. The sample was carefully washed with PBS and then incubated with PRP for 15 min at room temperature. After the reaction, the sample was thoroughly washed with PBS. The samples were denoted as PCL-COOH-PRP2.

### 2.5. Cell Tests

Human fibroblast cells (MRC-5 line) were purchased from the State Research Center of Virology and Biotechnology VECTOR (Novosibirsk, Russian). Cells were cultured in Dulbecco’s modified Eagle medium (DMEM, Sigma Aldrich, St. Louis, MO, USA) supplemented with 10% fetal bovine serum (Gibco) under standard culture conditions (humidified atmosphere, 5% CO_2_ and 95% air, at 37 °C).

#### 2.5.1. Adhesion Assay

Round-shaped scaffolds (PCL-ref, PCL-CCOH, and PCL-COOH-PRP2) with a diameter of 6 mm were placed in a 96-well plate. The cells were seeded on the scaffolds at a concentration of 10 × 10^3^ cells per scaffold. After 20 min (short-time adhesion), 2 and 24 h, and three and seven days, the scaffolds with fibroblasts were fixed using 4% paraformaldehyde solution for 10 min. Then, 0.1% Triton X-100 was used for the cell membrane permeabilization. For visualization of cytoskeleton actin filaments, we used the Alexa Fluor 532 phalloidin (Thermo Fisher Scientific, Waltham, MA, USA). The fibroblast-seeded scaffolds were stained with Hoechst 33342 for 15 min at room temperature. The cell adhesion on the PCL nanofibers was studied by fluorescence microscopy (Zeiss, Axio observer Z1, Oberkochen, Germany).

#### 2.5.2. Cell Proliferation

The Click-iT™ EdUAlexa Fluor™ 488 Imaging Kit (Thermo Fisher Scientific, Waltham, MA, USA) was used for detection of the cell proliferation rate. Each round-shaped scaffold with a diameter of 6 mm was seeded with the fibroblasts at a concentration of 7 × 10^3^ cells per well in the 96-well plates. The cell-seeded scaffolds were incubated under standard condition (5% CO_2_ and 95% air) at 37 °C. On the third and seventh day after seeding the cells, EdU was added to each sample for 2 h. The cells were studied by fluorescence microscopy (Zeiss, Axio observer Z1, Oberkochen, Germany). The cell quantification was performed on 50 microscopic images per studied scaffold. The level of proliferation rate was estimated by determining the ratio of total cells (Hoechst-positive) to the EdU-positive cells using the Cell Activision software (Yokogawa Electric Corporation, Tokyo Japan).

#### 2.5.3. Cell Apoptosis

The fluorescent dye Hoechst 33342 was used to stain cell nuclei. The percentage of apoptotic cells was evaluated as the ratio of normal cell nuclei (middle bright and uniformity of Hoechst) to fragmented and brighter nuclei.

#### 2.5.4. Immunofluorescent Staining of Scaffold Cells

Fibroblasts were grown on scaffolds for three and seven days. Afterward, they were fixed with 4% paraformaldehyde, permeabilized with 0.1% Triton X-100, and blocked with 1% BSA (bovine serum albumin). The cells seeded on the scaffold were stained with primary antibodies for 4 h at room temperature, washed with PBS, and incubated with secondary antibodies for 1 h at room temperature. The stained cells were analyzed with the fluorescence microscope (Zeiss, Axio observer Z1, Oberkochen, Germany).

The following primary antibodies were used: anti-fibronectin (Abcam, Cambridge, UK, ab6328, 1:200) and anti-collagen IV (Life Span, Hamilton, OH, USA, 1:200). The following secondary antibodies were used: Alexa Fluor 594 goat anti-mouse immunoglobulin G1 (IgG1; Life Technologies, Carlsbad, CA, USA).

All results were displayed as mean values and standard errors. The statistical significance was assessed by the nonparametric Mann–Whitney U test using the Statistica 10 software (StatSoft. Inc., Tulsa, Ok, USA).

## 3. Results

### 3.1. Optimization of Electrospinning of the PCL Nanofibers

**Figure 1:**
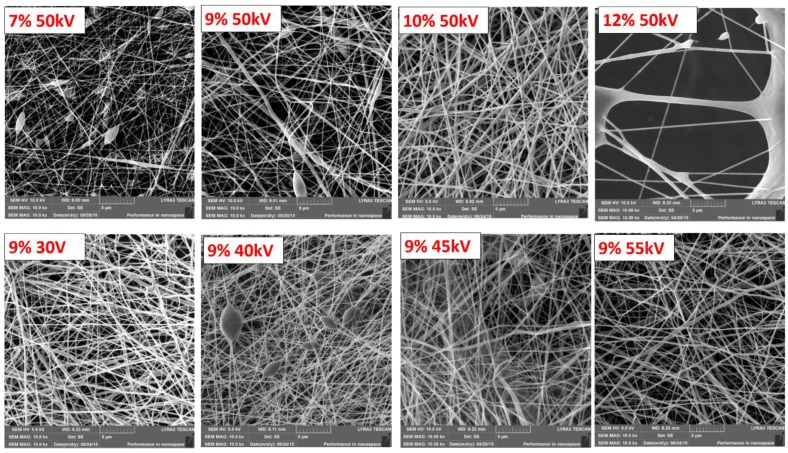
SEM micrographs of the polycaprolactone (PCL-ref) nanofibers obtained from the PCL solutions with the different PCL concentrations and at different voltages. The PCL concentration and voltage are reported on the images. The size of the bar corresponds to 1 µm.

The effect of the electrospinning process on the resulting polymer nanofibers was studied in order to obtain homogeneous substrates for subsequent immobilization and biological tests. The nanofibers’ topography analyzed by SEM is shown in Figure 1. The comparison of the micrographs reveals a highly porous network of fibers with considerable differences in morphology and homogeneity depending on the PCL concentration in solution and the applied voltage.

The effect of the PCL concentration on the electrospinning solution is shown in the upper row of images in Figure 1, captured at the magnification of 10,000×. The applied voltage was consistent for all substrates at 50 kV. The electrospun nanofibers prepared from the solution with 7 wt.% of the PCL polymer were thin with a calculated thickness of 132 ± 42 nm, and they exhibited a significant beaded structure. Beads and beaded fibers are more likely formed from less concentrated solutions, as their viscosities are low, making the jet formation unstable [36]. Increasing the PCL concentration led to an increase in fiber uniformity and a more regular morphology with a considerable decrease in beaded fibers [36,37].

**Figure 2:**
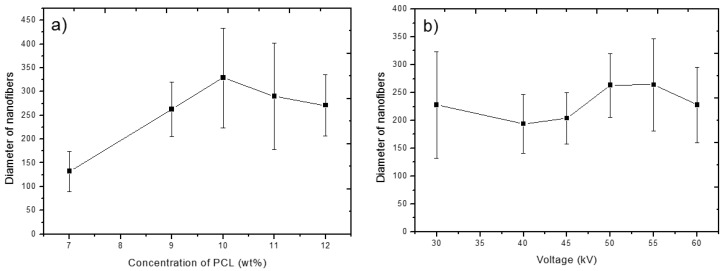
Thickness of resulting PCL nanofibers as a function of the PCL concentration (**a**) and applied voltage (**b**) during the electrospinning process. Error bars represent standard deviation of the nanofibers’ thickness distribution.

The nanofibrous meshes prepared from solutions with PCL concentrations of 9 and 11 wt.% gave fiber thicknesses of 263 ± 57 nm and 290 ± 112 nm, respectively. A higher thickness of nanofibers and their lower homogeneity prepared from more concentrated solutions might be caused by the viscoelastic force in the nanofiber jet resisting the stretching repulsive forces of opposing charges [38]. The effect of PCL concentration on the thickness of nanofibers is summarized in Figure 2a.

For further experiments, a PCL concentration of 9 wt.% was selected, and the effect of the applied voltage was studied. The SEM micrographs of the electrospun PCL nanofibers obtained with the applied voltages of 30, 40, 45, and 55 kV are shown in the lower row of images in Figure 1. The effect of applied voltage is summarized in Figure 2b. It can be seen that the effect of the applied voltage on the fiber diameter is not linear. It is worth noting that the extremely low values of applied voltage led to a higher dispersion of fiber diameters. Depending on the combination of process parameters, it is possible to obtain a substrate with fine homogeneous nanofibers.

The observed effect of polymer concentration and applied voltage on fiber thickness and homogeneity is summarized in Figure 2a,b. The error bars represent the calculated standard deviation of nanofiber thickness on selected substrates and, thus, illustrate their homogeneity. As visible in the graphs, a lower concentration of PCL polymer in solution (7 wt.%) led to thinner fibers. Nevertheless, because of the occurrence of beaded structures, the substrates were not used for further experiments. The increase in nanofiber thickness correlated with increasing PCL polymer concentration in solution and was less dependent on applied voltage. In addition, the nanofiber thickness was more homogeneous when less concentrated/viscous solutions were used.

**Figure 3:**
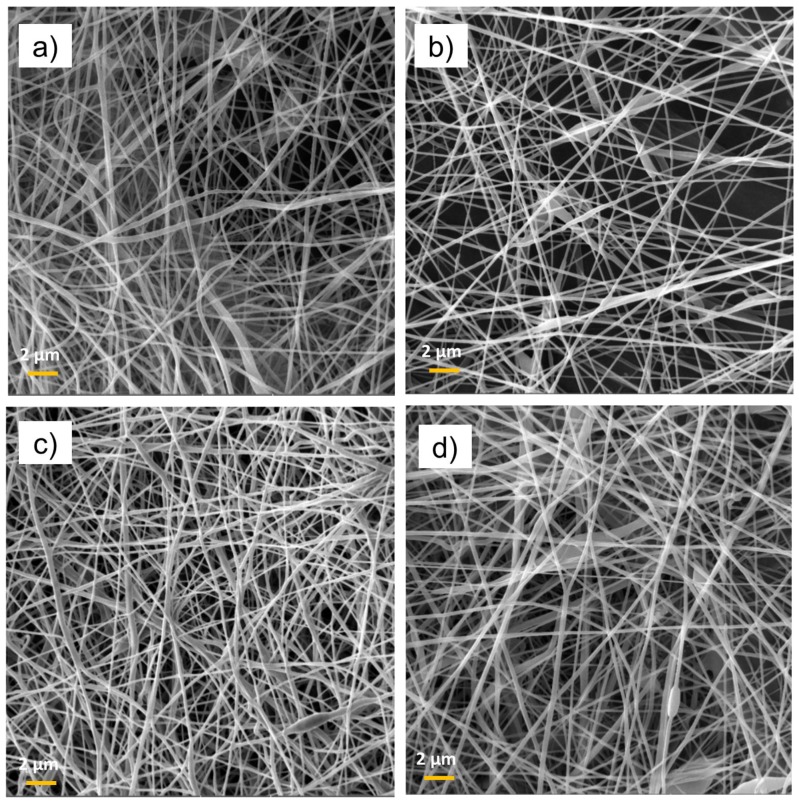
SEM micrographs of PCL-ref (**a**), PCL/platelet-rich plasma (PCL-PRP) (**b**), PCL-COOH (**c**), and PCL-COOH-PRP2 (**d**) The size of the bar corresponds to 1 µm.

The observed tendency is in line with the previously reported data for the optimization of electrospinning from a PCL solution in acetic acid using the Starter Kit 40 KV Web set-up, utilizing the needle electrospinning technology [36]. The advantage of needleless technology developed by Elmarco (used here) is a higher production rate and a bigger size of the produced samples. Regarding the optimized conditions, the polymer substrates electrospun from solutions with a PCL concentration of 9 wt.% and applied voltage of 50–55 kV were selected for plasma chemical depositions. From now on, the paper refers to this type of nanofiber simply as PCL-ref. The morphology of the nanofibers was not affected by the deposition of Ar/CO_2_/C_2_H_4_ plasma layers and no damage was detected (Figure 3). The immobilization of PRP also had no effect on the morphology of the PCL nanofibers. No swelling or degradation of fibers can be observed in the SEM micrographs in Figure 3.

### 3.2. Surface Characterization of the Samples

**Figure 4:**
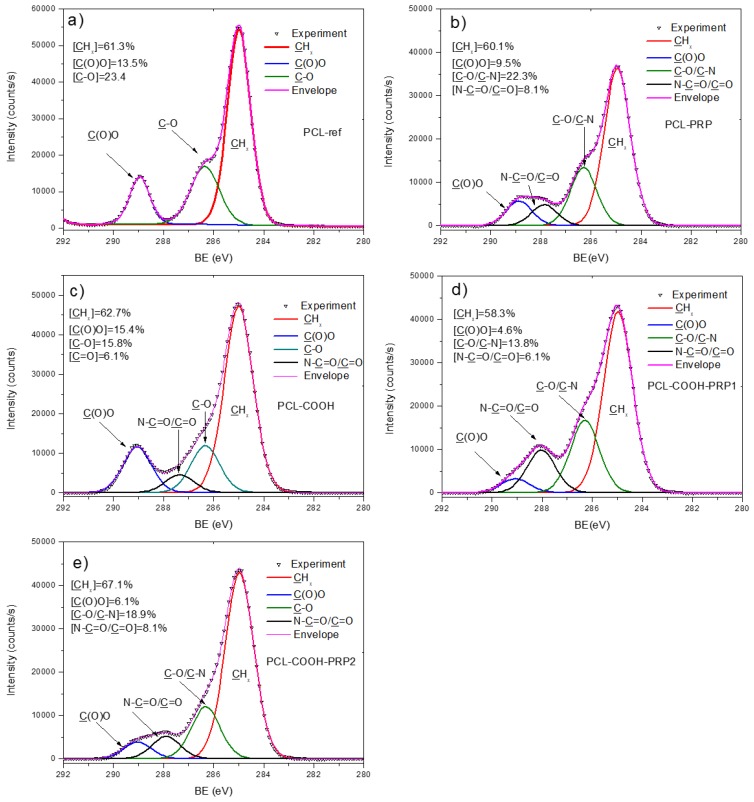
X-ray photoelectron spectroscopy (XPS) C *1s* curve fitting of PCL-ref (**a**), PCL-PRP (**b**), PCL-COOH (**c**), PCL-COOH-PRP1 (**d**), and PCL-COOH-PRP2 (**e**).

The effect of plasma coating was evident from the WCA measurements. PCL-ref exhibited a WCA of 121 ± 3°. The deposition of the Ar/CO_2_/C_2_H_4_ plasma layer led to a decrease in WCA to 67 ± 7°. The increase in wettability of PCL nanofibers is related to the grafting of polar groups and it was also shown before for COOH modification of PCL nanofibers using the atmospheric pressure plasma deposition technique [5]. The chemical composition of the PCL nanofibers (PCL-ref) measured by XPS is reported in Table 1. The atomic percentages of the elements were quantified from the high-resolution spectra of each element. The survey spectra of the samples are reported in Figure S1 (Supplementary Materials). The XPS C *1s* spectrum of PCL-ref can be fitted with a sum of three components, namely hydrocarbons CH_x_ (BE = 285 eV), ether group C–O (BE = 286.4 eV), and ester group C(O)O (BE = 289.0 eV) (Figure 4a). The full width at the half maximum (FWHM) of C–O was set to 1.35 eV, while CH_x_ and C(O)O were fitted with the FWHM of 1.1 and 0.95 eV, respectively. The XPS O *1s* spectrum was fitted with a sum of two components: C=O (BE = 532.1 eV) and C–O (BE = 533.5 eV). The relative concentrations of C=O and C–O components were 42% and 58%, respectively (see Figure S2, Supplementary Materials).

**Figure 5:**
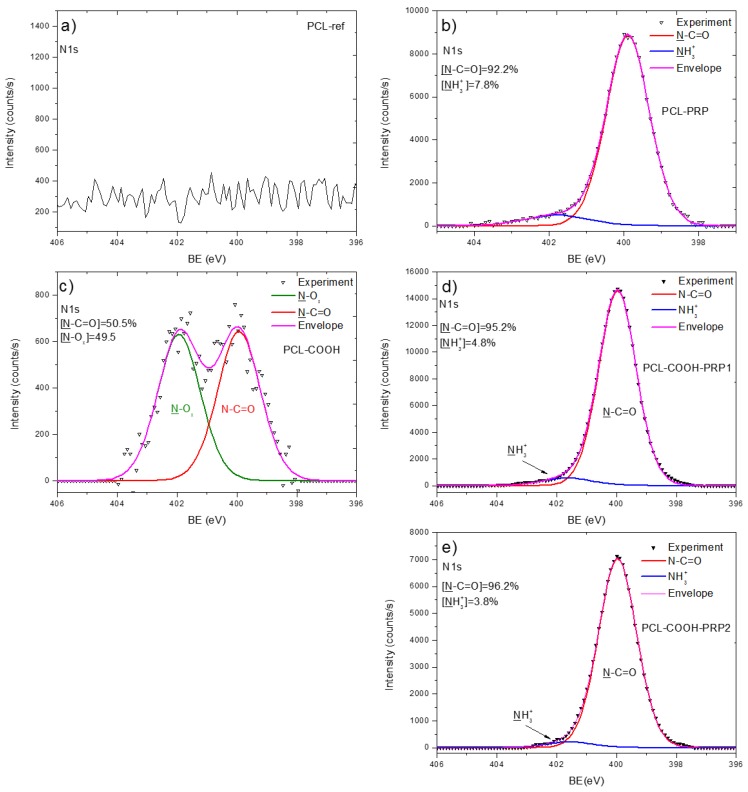
XPS N *1s* curve fitting of PCL-ref (**a**), PCL-PRP (**b**), PCL-COOH (**c**), PCL-COOH-PRP1 (**d**), and PCL-COOH-PRP2 (**e**).

The deposition of Ar/CO_2_/C_2_H_4_ plasma layers led to a slight increase in the O/C ratio. Interestingly, the observed changes in the XPS C *1s* spectrum were rather significant (Figure 4c). The XPS C *1s* spectrum of the Ar/CO_2_/C_2_H_4_ plasma-coated nanofibers (PCL-COOH) was fitted with four carbon components: CH_x_ (BE = 285 eV), C–O (BE = 286.4 eV), C=O (BE = 287.4 eV), and C(O)O (BE = 289.1 eV). The FWHM value for all components was set to 1.35 ± 0.05 eV. The concentrations of different carbon environments are summarized in Figure 4c. XPS analysis also revealed a small amount of nitrogen present on the PCL-COOH sample. The XPS N *1s* spectrum of PCL-COOH (Figure 5c) was fitted with two components: amide group N–C=O (BE = 400.0 eV) and N–O_x_ (BE = 402.0 eV). Most likely, the origins of these groups are related to the reaction with residual nitrogen in plasma or to the contact of PCL-COOH with the atmosphere. The O *1s* spectrum after the deposition of plasma layers was fitted with the same two components C–O and C=O. However, the FWHM of these peaks became broader and the concentration of the C=O component was increased by ~20% at the expense of the C–O component (see Figure S2, Supplementary Materials).

Concerning the changes in the surface chemistry after the immobilization, according to the XPS results in Table 1, it is evident that the immobilization of PRP happens even without the plasma coating of the nanofibers. It is indicated by the relatively high concentrations of nitrogen found on the PCL- PRP, PCL-COOH-PRP1, and PCL-COOH-PRP2 samples. It is worth noting that the traces of sodium (<1 at.%), chlorine (<1 at.%), and phosphorus (<0.5 at.%) were also detected on survey scans (see Figure S1, Supplementary Materials). These traces are the components of the PBS (NaCl and Na_2_HPO_4_) that we used for washing of the sample surface and, therefore, these elements were not taken into account in Table 1.

In addition, the ATR-FTIR analyses (not reported here) showed that there were no changes in the spectrum of PCL-PRP in comparison to the one of PCL-ref. The XPS N *1s* spectrum of PCL-PRP is presented in Figure 5b. The PCL-ref did not provide any N *1s* signal, whereas PCL-PRP, PCL-COOH-PRP1, and PCL-COOH- PRP yielded very similar spectra that were all fitted with a sum of two components, namely an amide group N–C=O (BE = 400.0 eV) and protonated amines NH_3_^+^ (BE = 401.7 eV). The FWHM for all components was set to 1.7 ± 0.1 eV. As shown in Figure 5b,d,e, for these samples, the main XPS N *1s* fit component is associated with amide groups, the building block of all proteins, thus confirming the immobilization of PRP. Interestingly, the highest concentration of nitrogen was detected for PCL-COOH-PRP1; this effect is most probably related to the steric hindrances induced by EDC activation as discussed in Section 4. It is worth noting that no unreacted EDC was detected on the surface, as no N=C=N component at 398.5 eV was found in the N *1s* spectrum. The XPS O *1s* spectra of PCL-PRP, PCL-COOH-PRP1, and PCL-COOH-PRP2 were fitted with a sum of C–O and C=O components (Figure S2, Supplementary Materials). Interestingly the non-covalent bonding of PRP led to the shift of the C=O component from 532.0 to 531.5 eV (in PCL-PRP and PCL-COOH- PRP1), whereas the covalent immobilization of PRP did not affect the BE position of this component. The shift is related to the different neighboring atom adjacent to the C=O component. The BE of 531.5 eV most probably corresponds to the C=O group close to amine group (NH), whereas the BE of 532.0 eV corresponds to C=O close to C–O (e.g., O–C=O).

### 3.3. Adhesion and Spreading of Fibroblasts Seeded on PCL-ref, PCL-COOH, and PCL-COOH-PRP

**Figure 6:**
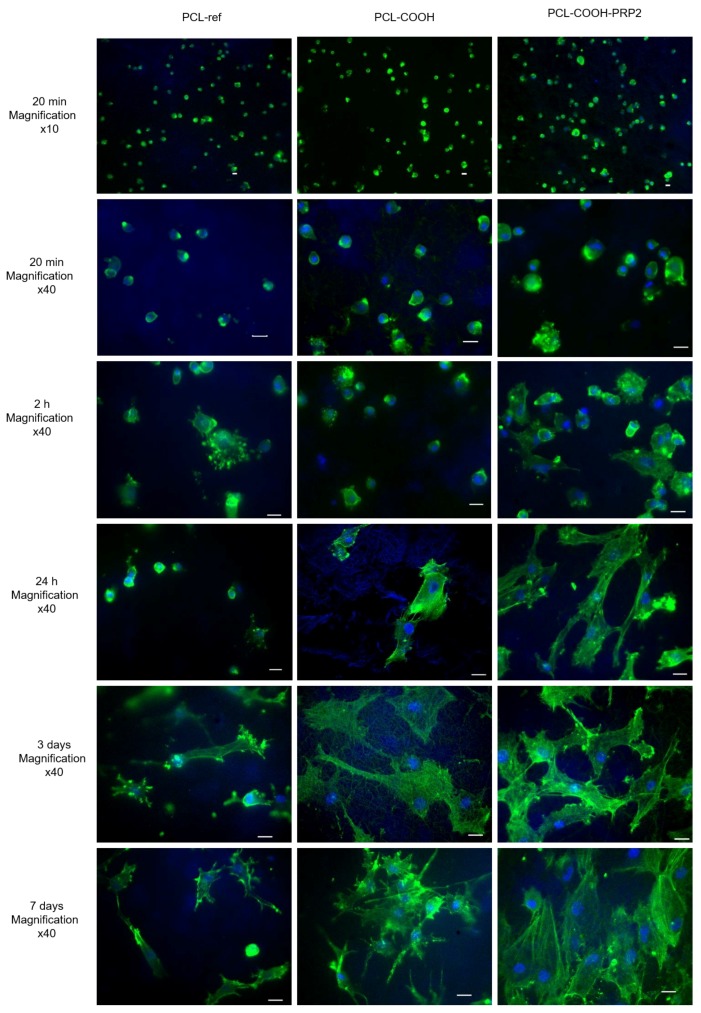
Fibroblast cell adhesion and spreading on the surface of PCL-ref, PCL-COOH, and PCL-COOH-PRP2 after 20 min, 24 h, and three and seven days of cultivation. The cytoskeleton actin filaments were stained by phalloidin (green), the cell nuclei were stained by Hoechst 33342 (blue). The size of the bar corresponds to 20 μm.

After 20 min of seeding cells on the studied scaffolds, the number of adhered cells showed no statistically significant differences. However, the largest cell size was found on PCL-COOH-PRP2 (up to 41 µm), while, on PCL-ref and PCL-COOH, the cell sizes reached a maximum of 35 µm (Figure 6).

After 2 h of cultivation, fibroblasts on all studied substrates formed filopodia. Nevertheless, as it can be seen from Figure 6, the number of cells on PCL-ref and on PCL-COOH was significantly lower compared to PCL-COOH-PRP2. Cells seeded on PCL-COOH-PRP2 had the biggest spreading area, multiple focal contacts, and filopodia, and a network of actin fibrils was formed (Figure 6 and Figure S3, Supplementary Materials).

After 24 h of incubation, the actin cytoskeleton of fibroblasts on PCL-ref was significantly pronounced at the edges of small rounded cells, whereas a network of actin fibers and stress fibrils was not observed. The fibroblasts adhered on the hydrophobic surface of PCL-ref, forming groups of cells.

The fibroblasts adhered onto PCL-COOH demonstrated a spread polygonal shape with a network of actin microfilaments. A part of the cells exhibited an elongated shape and stress fibrils (Figure 6). The fibroblasts were evenly distributed on the surface.

There was no significant difference in the number of cells after 24 h of incubation on PCL-ref and PCL-COOH. The binding of PRP to the substrate affected the adhesion of fibroblasts already at the initiation stage and, after one day, the cells had a well-spread polygonal shape with a pronounced cytoskeleton and lamellipodia (Figure 6).

Within three days, the number of adhered cells on PCL-ref did not increase significantly. The cells were of small size and exhibited multiple focal contacts. On the third day of incubation, the cells on PCL-COOH exhibited a relatively pronounced cytoskeleton and a flattened polygonal shape. However, the cytoskeleton was less pronounced than on PCL-COOH-PRP2 and there were no clear stress-fibrils and focal contacts. At this incubation time, the cells had a well-formed cytoskeleton, organized stress fibrils, focal contacts, and lamellipodia on PCL-COOH-PRP2.

On PCL-ref, the number of cells after seven days of incubation did not differ statistically from that after three days. The cells revealed cytoplasmic micro-growths directed anisotropically. On PCL-COOH, despite the statistically important increase in the cell number after seven days of incubation, the cells also exhibited long, anisotropically directed cytoplasmic micro-growths. (Figure 6). The increased adhesion on the PCL-COOH-PRP2 substrates during early and late spreading stimulated cell proliferation, as, after seven days of incubation, a monolayer of fibroblasts was observed (Figure 6 and Figure S3, Supplementary Materials). There was a contact inhibition of the isotropic movement and, thus, the cells were co-directed (Figure 6).

### 3.4. The Modification of PCL Nanofibers with COOH Groups and Its Effect on Human Fibroblast Proliferation and the Cell Apoptosis

**Figure 7:**
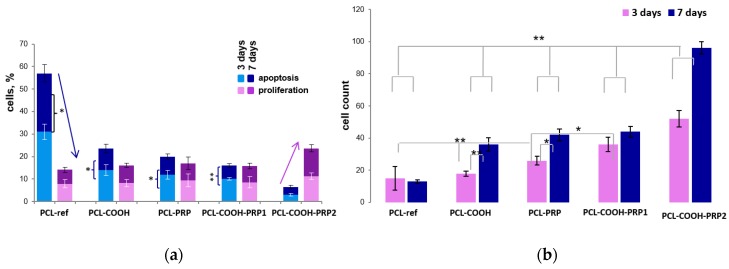
The influence of scaffold surface on (**a**) cell proliferation and apoptosis, and (**b**) cell count for three and seven days of cultivation. The level of proliferation rate was estimated by determining the ratio of total cells (Hoechst-positive) to the EdU-positive cells. The percentage of apoptotic cells was calculated as the ratio of the nuclei with chromatin condensation and nuclear fragmentation (kariorhexis) to nuclei with homogeneous coloration. The arrows indicate the relationship of the level of cell apoptosis and cell proliferation. Data are expressed as means ± standard errors; ** *p* < 0.01, * *p* < 0.05.

After 24 h of incubation, the number of adhered cells on PCL-ref and PCL-COOH did not differ significantly; however, on PCL-COOH, the cells were distributed more evenly. The number of cells on the third day of incubation on PCL-ref and PCL-COOH scaffolds made up 15 ± 7.4 and 18 ± 4.2 cells per field of view, respectively (Figure 7b). The level of cell proliferation on PCL-ref and PCL-COOH was not statistically different and was equal to 7.9 ± 1.9% and 8.2 ± 1.6%, respectively (Figure 7a). The level of apoptosis on PCL-ref was high (31 ± 3.4%), and that on PCL-COOH was two times lower (14 ± 2.3%) (Figure 7a).

Due to the uneven density of cells on PCL-ref, the fields of view were divided into two regions with a high and low cell content. The level of proliferation was highly dependent on the cell number on the PCL-ref substrates. Thus, 10% EdU-positive cells were in the regions with a high cell density, while a low cell density demonstrated 4.5% proliferating cells.

At the same time, on PCL-COOH, the proliferation level was practically independent of the number of cells per field.

On the seventh day of incubation, the average number of cells on PCL-ref and PCL-COOH made up 13 ± 1.1 and 36 ± 4.2 cells per field of view, respectively (Figure 7b). The proliferation level on PCL-ref and PCL-COOH substrates did not change statistically. At the same time, the number of apoptotic cells seeded on PCL-ref and PCL-COOH decreased to 26 ± 3.9% and 9.5 ± 1.9%, respectively (Figure 7a). The reduced apoptosis was probably due to the production of the extracellular matrix by viable cells.

Therefore, the COOH modification of the PCL nanofibers promotes a more uniform distribution of adhered cells and a better cell viability associated with a decrease in the level of apoptotic cell death, suggesting a better biocompatibility of this surface as compared to unmodified PCL nanofibers. The fibroblasts are capable of adhering and surviving on the hydrophobic surface of PCL-ref, maintaining a certain level of proliferation in the islands of high cell density.

### 3.5. The Effect of PRP Immobilization on Human Fibroblast Proliferation

We analyzed the proliferation of human fibroblasts on the PRP-modified PCL nanofibers with different forms of PRP bonding: non-covalent bonding of PRP to unmodified PCL-ref based on hydrophobic–hydrophobic interaction of the proteins with PCL nanofibers, ionic bonding of PRP to COOH group plasma-coated PCL scaffolds, and covalent bonding of PRP to the PCL-COOH using EDC.

Observation over three days showed that the addition of PRP to PCL significantly increased the number of cells. It was noted that the number of cells on PCL-PRP and PCL-COOH-PRP1 was lower compared to PCL-COOH-PRP2 (26 ± 2.6 and 32 ± 4.4 against 52 ± 5, respectively). The level of proliferative activity was also higher for PCL-COOH-PRP2, at 11.2 ± 1.5%, compared with 8.6 ± 1.32% and 9.4 ± 2.9% for PCL-COOH-PRP1 and PCL-PRP, respectively (Figure 7).

**Figure 8:**
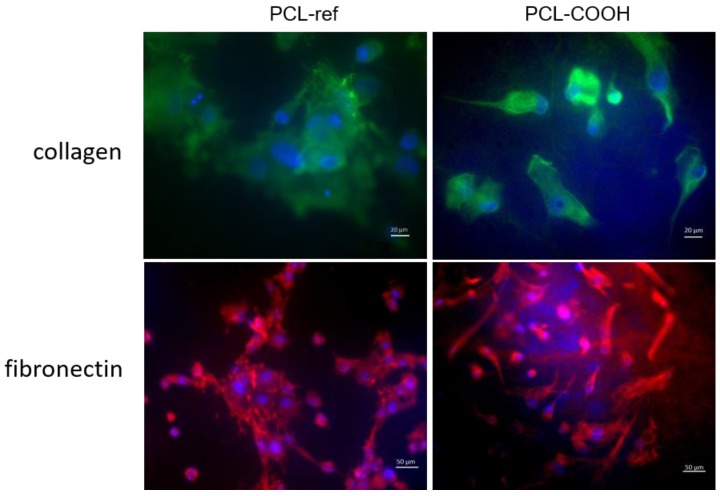
Fibronectin (FN) and collagen secretion by the fibroblasts seeded on PCL-ref and PCL-COOH. Cells were stained by antibody Alexa Fluor 594 (orange) to collagen (magnification 40×) and Alexa Fluor 488 to FN (magnification 20×).

It should be noted that the level of apoptosis on PCL-COOH-PRP2 was three times lower compared to PCL-COOH-PRP1 and PCL-PRP (3 ± 0.7% versus 10 ± 0.6% and 12 ± 1.9%, respectively) (Figure 8). Thus, already on the third day, it can be seen that the surface with covalently bound PRP was most effective for maintaining the functional viability of fibroblasts.

After seven days on PCL-COOH-PRP1, the level of cell apoptosis decreased to 6 ± 0.9%, while maintaining cell proliferative activity (7.2 ± 1.3%). The percentage of apoptotic cell death on PCL-COOH-PRP2 remained at the same low level (3.5 ± 0.7%), while maintaining the level of proliferative activity of 12.4 ± 1.6%.

Thus, it is shown that the addition of PRP to PCL-ref and PCL-COOH nanofibers further increased the number of adhered cells and the level of cell proliferation, and decreased the level of apoptotic death. However, hydrophobic non-covalent and ionic bonding of PRP to PCL-COOH and PCL-ref led to a weak linkage between proteins and PCL and, as a result, only a short-term enhancement of the cell proliferation (Figure 7). The dynamics of the cell number growth and percentage of proliferating cells reduced after three days of cultivation. The covalent bonding of PRP to PCL-COOH ensured a constantly enhanced level of proliferation and a low level of fibroblast apoptosis even after seven days of cultivation.

### 3.6. The Secretion of Fibronectin and Type IV Collagen by Fibroblasts Seeded on PCL-ref

As shown earlier, the uneven cell distribution on PC-ref was associated with a high single-cell death and a good level of proliferation in the islands of high cell density. The cells on PCL-ref had a normal phenotype and a high expression level of fibronectin (FN) and collagen after three days of incubation in the areas of high cell density. On PCL-COOH, the fibroblasts were located evenly and secreted FN and collagen (Figure 8).

This pattern of ECM component secretion by fibroblasts also suggests that PCL-COOH is more suitable for cell viability.

## 4. Discussion

For the purpose of tissue engineering, as well as for regenerative medicine, it is necessary to create scaffolds suitable for the viability and functional activity of the cells [39]. The aim of this study was to create a nanofibrous scaffold suitable for the efficient cell adhesion, spreading and functional activity of fibroblasts. The normal cell adhesion and spreading on the substrate surface affect the later stages of the cell functional activity [14,15,16,17]. Therefore, the study of cell spreading is vital in the scaffold’s screening for the purpose of regenerative medicine. The initial stage of cell adhesion is determined by the physico-mechanical interactions of the physical body (cell) and the substrate, and it does not depend on the molecular integrin–ECM interactions [15]. It is characterized by the blabbing of rounded cells, formation of a smooth contact area, and passive attachment to the substrate, and it lasts for 5–20 min after the cell seeding. We do not observe differences at this stage neither in the number nor in the morphological features of cells on the studied PCL scaffolds 20 min after the cell seeding (Figure 6). However, the bigger size of some of the cells on PCL-COOH-PRP2 was a result of better adhesion and an earlier spreading of fibroblasts.

The longer phase of early spreading lasted for approximately 120 min and was defined by the active cell attachment with the organization of a peripheral zone by actin fibrils and the formation of lamellipodia. At this phase, the polymerization of actin and the contraction of myosin are necessary for the polarization and movement of cells.

This phase was significantly extended until 24 h for PCL-COOH and until three days for PCL-ref. The electrophilicity of the PCL-COOH surface, together with the negative charge of the cells, led to a delay in their interaction. The presence of divalent cations in the cell culture medium of the PCL-COOH surface allowed the cells to adhere faster as compared to the hydrophobic surface of PCL-ref. The delay in the initial stage of adhesion and spreading on PCL-ref and PCL-COOH affected the proliferation rate and cell viability in the future and reduced the efficiency of PCL nanofiber colonization by fibroblasts (Figure 6 and Figure S3, Supplementary Materials).

It was previously shown that mesenchymal stem cells (MSCs), secreting mainly trophic factors, had good spread area on PCL-ref hydrophobic surfaces during the first hours after seeding but they did not survive on the PCL-ref surfaces during further cultivation [40]. Even though MSCs have highly promising regenerative potential, numerous MSC transplantation experiments showed that cells have a transient paracrine effect and do not survive in the pathologic area, losing their regenerative potential [41,42,43]. Fibroblasts have a morphology similar to MSCs and express a number of mesenchymal markers (cluster of differentiation 90 (CD90), CD105, CD44, and CD73). However, secretion of ECM components is the main function of these cells. In this work, the fibroblasts secrete ECM components, enriching PCL nanofibers with FN and collagen, thereby increasing the biocompatibility of the PCL-ref hydrophobic surface. On the third day of incubation, the fibroblasts cultivated on PCL-ref were found out to actively secrete FN and collagen in the cell islands. This promoted cell survival and proliferation, while the single cells died. Throughout the entire experiment, a high level of the fibroblast anoikis on PCL-ref was observed due to the surface adhesion deterioration [44] (see Figure 6 and Figure S3, Supplementary Materials). However, the observation of cells after seven days of cultivation revealed that the cells detached from the PCL nanofibers, despite the increase in cell density in the cell “islands” and the active secretion of fibronectin (data not shown). This can be explained by the fact that poor initial cell attachment to the substrate leads to the fibronectin turnover shift to the intercellular contacts instead of the cell–matrix contacts [29,45]. The increased cell-to-cell contact creates an area of an increased tension and leads to the cell detachment from the substrate [46].

The simple soaking of PCL-ref in PRP solution (non-covalent bonding) enabled immediate cell attachment to the substrate, thereby increasing the possibility of normal functioning of fibroblasts in the future. The grafting of PRP onto the uncoated PCL nanofibers can be explained by the interaction of the hydrophobic shell of proteins with the hydrophobic nanofibers. Such a process can potentially happen only on the very top surface of the fibers and it would lead to a very small immobilization of PRP. This was confirmed by the shape of the XPS C *1s* spectrum exhibiting many similarities to the spectrum of uncoated nanofibers (see Figure 4a,b). However, leaching of the PRP components from the surface of the nanofibers during cultivation resulted in a bare PCL-ref hydrophobic surface, leading to an increase in the anoikis of the cells.

In order to improve the biocompatibility of PCL nanofibers, an additional modification of their surface by the binding of freeze–thawed platelet-rich plasma was used.

The XPS data revealed that, after the immobilization of PRP, there was 0.2 at.% sulfur in the layer and the N *1s* signal consisted of N–C=O and NH_3_^+^. When compared to literature data for FN XPS analysis, the BE of the S *2p* peak, as well as the shape of the N *1s* peak, was very similar to the reported ones [47].

**Figure 9:**
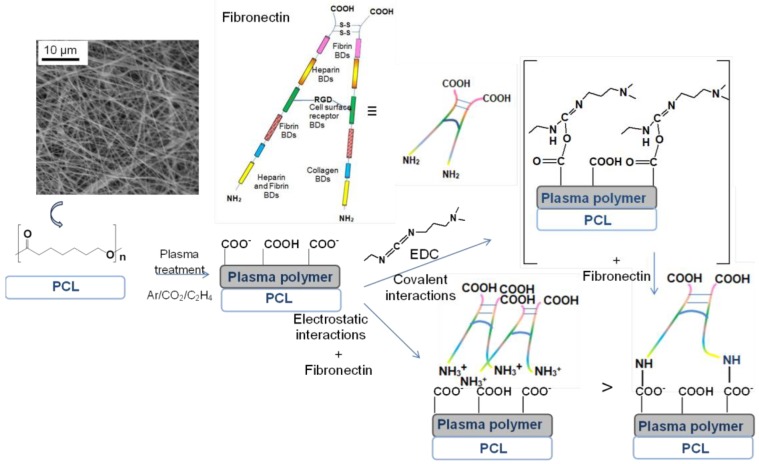
Scheme of “electrostatic” and covalent immobilization of FN on PCL-COOH.

In order to understand how bioactive molecules attach to the PCL nanofibers, it can be useful to look at the immobilization process using the example of FN immobilization. When considering the immobilization of FN on the PCL nanofibers, one needs to remember that FN has a fibrillary structure with NH_2_ and COOH groups at the “ends” of the long fibrils. In the absence of COOH groups on PCL nanofibers, the only possible binding of FN to PCL-ref would be the non-covalent binding via the interaction of the hydrophobic parts of FN with the hydrophobic PCL nanofibers. Only one monolayer of proteins can be immobilized on the top surface of PCL-ref and, certainly, the bonding will be very weak. The immobilization of FN directly on PCL-COOH (without EDC activator) would lead to the electrostatic coupling of FN to PCL-COOH, as shown in Figure 9. The absence of covalent bonding, a relatively long aliphatic chain between the “body” of protein and the amine group, and the excess of proteins in the PRP solution allow all available sites on the PCL-COOH-PRP1 to become “freely” occupied. The activation of the COOH groups by EDC leads to the grafting of carbodiimide bearing bulky fragments (see Figure 9). Most probably, these groups occupy more space on the surface during interaction in comparison to the amine group in the long aliphatic chain at the FN end. As a result, the concentration of nitrogen on PCL-COOH-PRP1 is higher by a factor of two than on PCL-COOH-PRP2. Hence, the density of the immobilized FN is higher for PCL-COOH-PRP1, but a weaker electrostatic bonding leads to the fast release of bioactive molecules, leaving the PCL-ref surface bare and with a hydrophobic nature. The formation of an area on the surface free of PRP molecules leads to a decrease in the survival of cells, thus increasing the apoptosis on the PCL-COOH-PRP1 and PCL-PRP samples (Figure 6).

Regardless of the type of bonding with PCL, the immobilization of PRP to PCL-ref and PCL-COOH leads to the normal cell adhesion and spreading due to the high biological activity of PRP components. The immobilization has a positive effect on the proliferative activity of fibroblasts and reduces the cell death by anoikis. However, the covalent bonding of PRP to PCL-COOH-PRP2 not only promotes the early stages of cell adhesion and spreading, but also significantly reduces the degree of cell apoptosis as compared to PCL-PRP and PCL-COOH-PRP1.

Most likely, the covalent bonding of PRP occurs over the entire volume of the porous plasma polymer coating and provides a stable fixation of biologically active substances of PRP, causing the consistently high cell proliferation rate throughout the study. Additionally, the confocal layer-by-layer imaging of fibroblasts seeded on PCL showed that the cells fill the entire thickness of the nanofibers, which, in this case, became “friendly for the cell”. The use of such matrices with covalently immobilized PRP for wound healing prolongs the biological activity of the immobilized molecules and protects them from the aggressive media of the wound. The proteins with a weak, non-covalent bonding to the PCL surface will be quickly washed out from the surface by exudation of the wound, leaving the PCL-ref surface bare and with a hydrophobic nature. Such a hydrophobic surface is not “cell-friendly” as discussed above.

Considering that the modification with COOH groups allows any type of proteins to be covalently attached, such a technique considerably widens the potential of the PCL nanofiber application in tissue engineering.

## 5. Conclusions

The reported modification of PCL nanofibers with COOH groups and the subsequent covalent binding of protein molecules is a rather simple and technologically accessible procedure. It was demonstrated that covalent binding of PRP occurs without the loss of its biochemical activity, allowing a significantly reduced fibroblast apoptosis and an increased cell proliferation on PCL-COOH-PRP2 compared with PCL-ref, PCL-COOH and PCL-COOH-PRP1 to be obtained.

The reported research findings reveal the potential of PCL matrices for application in tissue engineering, while the plasma modification with COOH groups and their subsequent covalent binding with NH_2_ groups of proteins expand this potential.

## Figures and Tables

**Table 1:** Atomic composition (at.%) of samples derived from X-ray photoelectron spectroscopy (XPS) analysis. The traces of sodium, chlorine, and phosphorus were detected but were not taken into account. PCL—polycaprolactone; PRP—platelet-rich plasma.

Sample Name	C	O	N	S
PCL-ref	73.9	26.1	0.0	0.0
PCL-PRP	71.6	20.6	7.6	0.2
PCL-COOH	72.4	26.7	0.9	0.0
PCL-COOH-PRP1	71.3	18.5	10.0	0.2
PCL-COOH-PRP2	75.8	18.1	5.9	0.2

## Supplementary Materials

The following are available online at https://www.mdpi.com/2079-4991/9/4/637/s1: Figure S1: XPS survey spectra of PCL-ref, PCL-PRP, PCL-COOH, PCL-COOH-PRP1 and PCL-COOH-PRP-2; Figure S2: XPS O 1s curve fitting of PCL-ref, PCL-PRP, PCL-COOH, PCL-COOH-PRP1 and PCL-COOH-PRP-2; Figure S3: The influence of dynamic disturbance of cell adhesion and spreading on cell proliferation. Representative images of cell adhesion and spreading stage on PCL-ref and PCL-COOH-PRP after 20 min and 2 hours. The second stage of adhesion (2 hours) is significantly slower on PCL-ref. The cells have the same morphology as on the early stage of adhesion (20 min). The cells seeded on PCL-COOH-PRP have a well-spread polygonal shape with a pronounced cytoskeleton and lamellipodia. As a consequence, the level of cell proliferation and the number of cells are the highest for PCL-COOH-PRP.
