# Supplementary material for: Plasma-Coated Polycaprolactone Nanofibers with Covalently Bonded Platelet-Rich Plasma Enhance Adhesion and Growth of Human Fibroblasts
Source: Nanomaterials (Basel). 2019 Apr 19;9(4):637. doi: 10.3390/nano9040637 (PMC6523319; doi:10.3390/nano9040637)
Supplement: Supplementary file 1 [file nanomaterials-09-00637-s001.pdf]

nanomaterials

Supplemental Information for the article

# Plasma-Coated Polycaprolactone Nanofibers with Covalently Bonded Platelet-Rich Plasma Enhance Adhesion and Growth of Human Fibroblasts

**Svetlana Miroshnichenko [1,2], Valeriia Timofeeva [1], Elizaveta Permykova [1,3], Sergey Ershov [4], Philip Kiryukhantsev-Korneev [3], Eva Dvořaková [5], Dmitry V. Shtansky [3], Lenka Zajíčková [5] Anastasiya Solovieva [1,*] and Anton Manakhov [1,*]**

1 Scientific Institute of Clinical and Experimental Lymphology—Branch of the ICG SB RAS, 2 Timakova str., 630060 Novosibirsk, Russian; svmiro@yandex.ru (S.M.); leravalera0204@mail.ru (V.T.); permyakova.elizaveta@gmail.com (E.P.)
2 Institute of Biochemistry – subdivision of the FRC FTM, 2 Timakova str., 630117 Novosibirsk, Russian
3 Laboratory of Inorganic Nanomaterials, National University of Science and Technology "MISiS", Leninsky pr. 4, 119049 Moscow, Russia; kiruhancev-korneev@yandex.ru (P.K.-K.); shtansky@shs.misis.ru (D.V.S.)
4 Physics and Materials Science Research Unit, Laboratory for the Physics of Advanced Materials, University of Luxembourg, 162a, avenue de la Faïencerie, L-1511 Luxembourg, Luxembourg; sergey.ershov@uni.lu
5 CEITEC—Central European Institute of Technology—Masaryk University, Kamenice 5, 625 00 Brno, Czech Republic; evke.dvorakova@gmail.com (E.D.); lenkaz@physics.muni.cz (L.Z.)
* Correspondence: solovey_ao@mail.ru (A.S.); ant-manahov@ya.ru (A.M.); Tel.: +7-913-906-0098 (A.S.); Tel.: +7-915-849-4059 (A.M.)

Received: 11 March 2019; Accepted: 15 April 2019; Published: 19 April 2019

**Content:**

1. XPS survey scans of the samples
2. XPS O *1s* curve fitting
3. The influence of dynamic disturbance of cell adhesion and spreading on cell proliferation. Representative images of cell adhesion and spreading stage on PCL-ref and PCL-COOH-PRP after 20 min and 2 h.

**XPS survey scans of the samples**

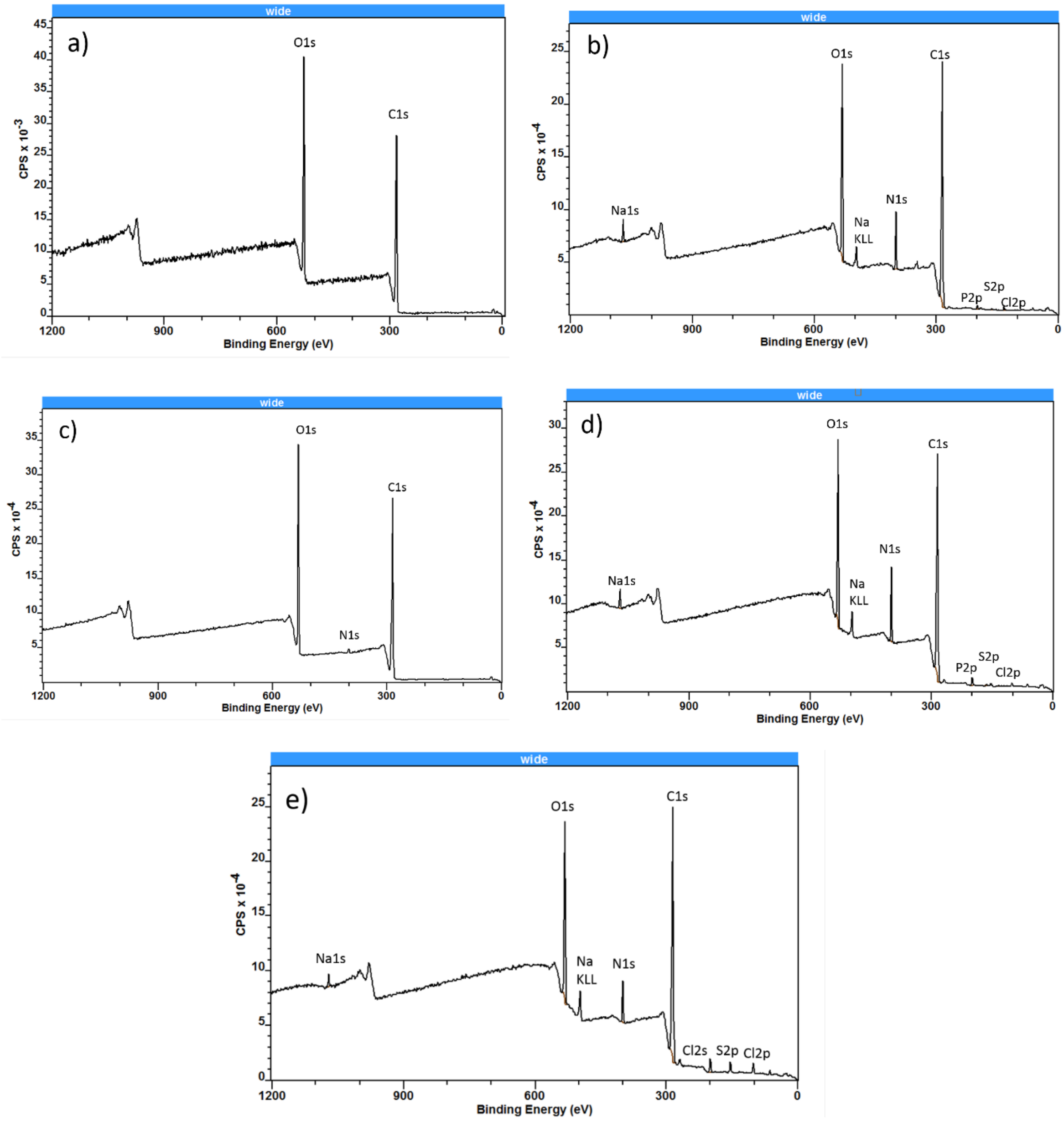


**Figure S1.** XPS survey spectra of PCL-ref (**a**), PCL-PRP (**b**), PCL-COOH (**c**), PCL-COOH-PRP1 (**d**) and PCL-COOH-PRP-2 (**e**).

The traces of sodium (<1 at.%), chrorine (<1 at.%) and phosphorus (<0.5 at.%) were also detected on survey scans (see Figure S1 in Supporting Information). These traces are related to the PBS washing of the samples and, therefore, were not takes into account in the Table 1.

## XPS O1s curve fitting

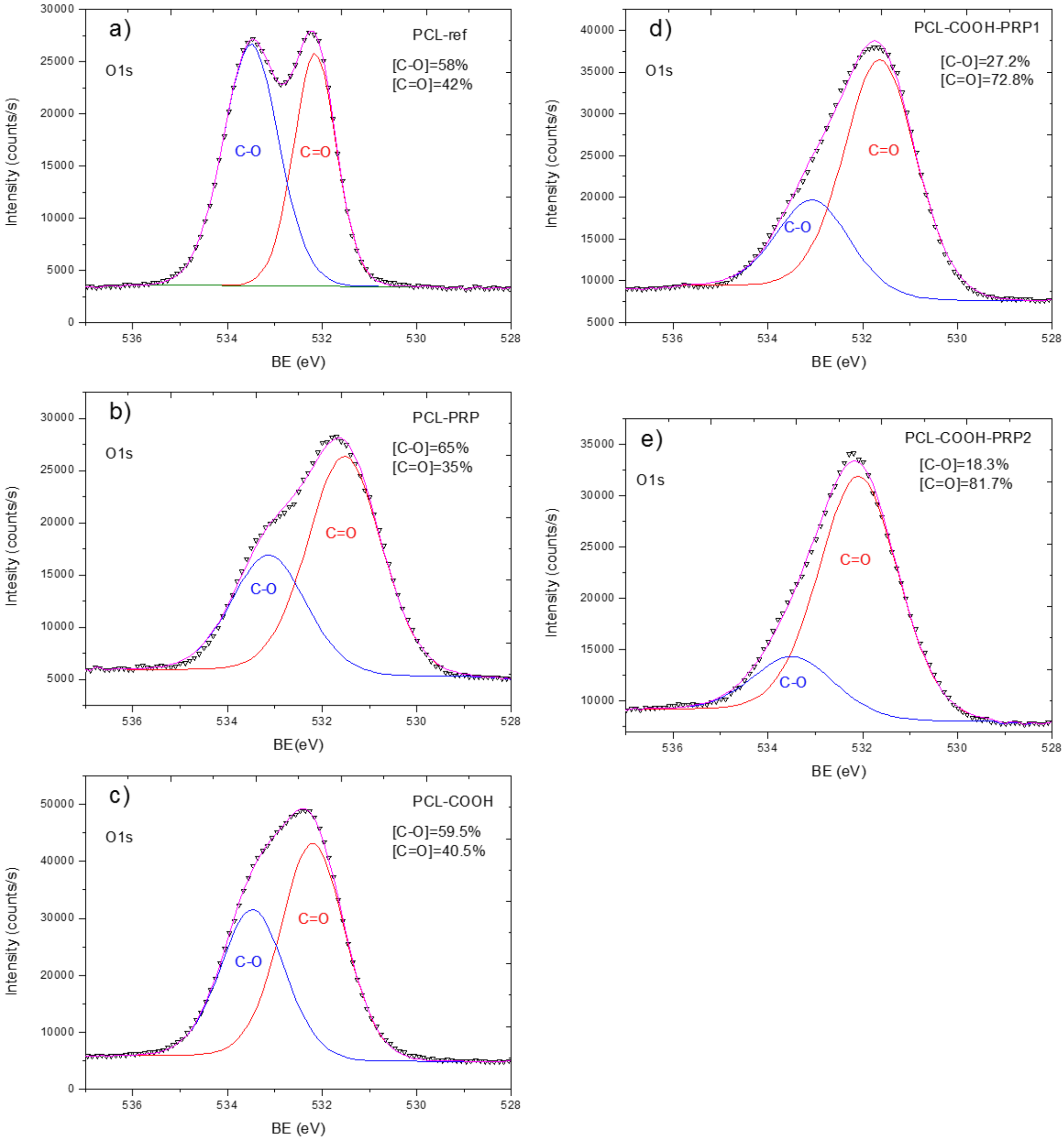


**Figure S2.** XPS O 1s curve fitting of PCL-ref (**a**), PCL-PRP (**b**), PCL-COOH (**c**), PCL-COOH-PRP1 (**d**) and PCL-COOH-PRP-2 (**e**).

The influence of dynamic disturbance of cell adhesion and spreading on cell proliferation. Representative images of cell adhesion and spreading stages on PCL-ref and PCL-COOH-PRP after 20 min and 2 h.

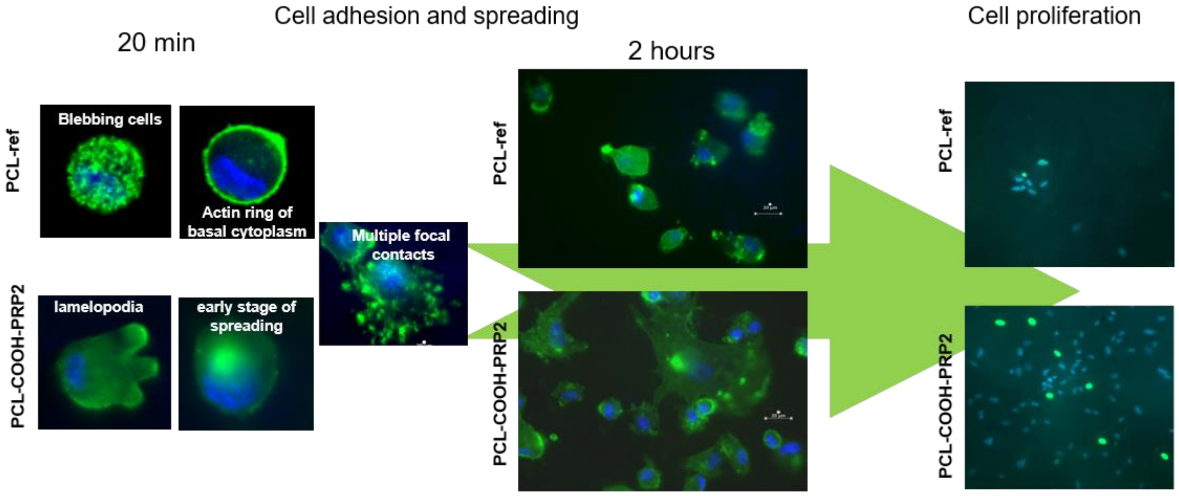


**Figure S3.** The influence of dynamic disturbance of cell adhesion and spreading on cell proliferation. Representative images of cell adhesion and spreading stage on PCL-ref and PCL-COOH-PRP after 20 min and 2 hours. The second stage of adhesion (2 hours) is significantly slower on PCL-ref. The cells have the same morphology as on the early stage of adhesion (20 min). The cells seeded on PCL-COOH-PRP have a well-spread polygonal shape with a pronounced cytoskeleton and lamellipodia. As a consequence, the level of cell proliferation and the number of cells are the highest on PCL-COOH-PRP. The cell nuclei were stained with Hoechst 33342 (blue) while the nuclei of proliferation cells were stained with Edu (green).
